# Importance of Dyspepsia

**Published:** 1894-03

**Authors:** Hugh Mathewson Patton

**Affiliations:** Montreal, Canada


### Importance of Dyspepsia.

BY HUGH MATHEWSON PATTON, M.D., MONTREAL, CANADA.

It has been estimated that nine-tenths of the cases of heart disease, kidney disease and rheumatism have origin in the faulty assimilation of the food ; the remaining tenth finding their source in the misuse of alcohol, accident and exposure. This leaves out heredity, and presupposes the individual to have started life with a set of organs in working order. So that if the physician will bend his energies to the correction of the habits which lead to indigestion, before organic disease superimposes simple deranged function, he will, in a large proportion of cases, restore his patient to the comfort ensuing from smoothly-working organs, and ward off organic disease in the organs noted above.

Crossing over a few days ago from Ireland, a prominent Montrealer asked my advice in regard to what he spoke of as swelling in his feet. He was a magnificent specimen of a man, but the slight bluish tinge of his lips and florid cheeks pointed to defective venous return. I examined him carefully, found slight edema of lower extremities, heart sounds normal, but very faint. A condition which I have learned by experience results in organic heart disease in a very short time.

I questioned him carefully in regard to his eating, and he assured me that no one could be more careful. I gave him nux vom. 2x before meals, and digitalis two drop doses, between meals, but the improvement was very slight.

Coming up the river I sat near him, and observed that while he carefully abstained from so-called indigestible foods, that what he did take was chewed two or three times and then *bolted* down by muscular force.

When I next saw him I mentioned this habit, and told him plainly that he would develop organic heart disease unless his food was properly eaten. He has wisely followed the suggestions, with a result that the dropsy has gone and his face assumed a normal color.

Just as an engine and boiler will get clogged up and work inefficiently, if the fuel is not properly burned, so will the human system get clogged up and work badly if the fuel is not properly assimilated. Hence, before I start to load a man up with drugs, I find out if he is giving the digestive juices of his body a fair chance.

I insist most strongly on a man eating at one time and drinking at another. If he takes his food dry, he must perforce chew it thoroughly. If he drinks when his stomach is empty, there is no waste of digestive juices, and the liquid which is not absorbed to meet the liquid needs of the tissues tends to waste away the catarrhal mucus which coats every disordered stomach and intestine.

To remove as far as possible the tendency to thirst at meal time I ask him to drink a glass of hot water, or hot milk and water one hour before each meal and bed time. To give the saliva a chance to digest the starchy elements of his food I ask him to chew his food so thoroughly that it will slip down his throat without effort.

The constant use of tea and coffee is on the same level with the constant use of alcohol—all admirable stimulants and adjuvants when used occasionally, but creating a craving habit, if constantly taken.

I advise him a good hour's walk daily, and morning and evening a five minutes' easy seance with the Goodyear Pocket Gymnasium, manufactured by Mr. Leonard, of Hodgman Brothers, Broadway.

The drugs which I have found were useful are carbo. veg. 5x, pulsatilla 3x, mercurius sol. 3x, bryonia 3x, hepar sulphur 5x, sulphur 6x, arsenic 5x, and cinchona 3x.

---

BICYCLE riding has proved curative in several cases of persistent sciatica.

---

THE item which is going the rounds quoting Dr. Lawson Tait as saying, "I pay not the slightest regard to sterilization of any kind; the whole of such precautions are farcial," reminds one of the reputed saying of Mark Twain, "I believe so thoroughly in total abstinence that I totally abstain from total abstinence itself." One of our leading operators in Chicago used to make fun of the antiseptic precautions of his confrere, but was in his own person as well as in his care of his instruments absolutely clean. After all, what is sterilization but scientific cleanliness? He who is clean in person, clothing, and instruments has given his patient a good chance for recovery—"A rose by any other name would smell just as sweet." Progressive surgery will continue to exercise due care, and he who knows how to do the best will reap the reward. Medicine has her Renans and Voltaires quite as much as religion, for both go on winning new laurels and increasing the armament of defence against the sneers of poticism.

